# Evaluation of cut-off values in acute paracetamol poisoning for safe termination of N-acetylcysteine

**DOI:** 10.1186/s40360-025-01075-y

**Published:** 2025-12-27

**Authors:** Jeeyong Lim, Kyungman Cha

**Affiliations:** 1 Department of Emergency Medicine, Seoul St. Mary’s Hospital, College of Medicine, The Catholic University of Korea, Seoul, Republic of Korea; 2 Department of Emergency Medicine, Suwon St. Vincent Hospital, 93 Jungbu Blvd, Paldal, Suwon, Gyeonggi 16247 Republic of Korea; 3 College of Medicine, The Catholic University of Korea, Seoul, Republic of Korea

**Keywords:** Paracetamol, Overdose, Acetylcysteine, Antidote

## Abstract

**Background:**

The Rumack-Matthew nomogram has been utilized to predict hepatotoxicity from acute paracetamol poisoning. Where paracetamol concentrations are unavailable, the commencement and cessation of treatment rely on reported dose. This study aimed to investigate risk factors predicting detection of paracetamol after 15 h.

**Methods:**

A retrospective analysis was conducted at two emergency centers from 2010 to 2020. Patients aged ≥ 14 years who ingested ≥ 75 mg/kg within 15 h were included. Exclusion criteria were chronic liver disease, taking multiple doses, sustained-release formations, or activated charcoal administration. Multinomial logistic regression was used to assess risk factors for detection of paracetamol after 15 h, and the area under the curve was calculated.

**Results:**

Among one hundred and ninety-four patients, 30 patients (15.4%) had detectable paracetamol and 7 patients (3.6%) showed toxic concentration after 15 h, and median ingested dose was 152.8 mg/kg. Time to presentation was significant for toxic concentration (Odds ratio = 1908), the area under the curve was 0.969, 612 min cut-off. Ingested dose and elevated liver enzyme were valid for detectable concentration (Odds ratio = 2.118 and 4.458), the area under the curve of ingested dose was 0.633, 105.2 mg/kg cut-off.

**Conclusion:**

Where paracetamol concentrations are unavailable and the United Kingdom guideline followed, for patients ≥ 14 years who present within 15 h and report ingestion ≥ 75 mg/kg, maximum therapeutic dose, N-acetylcysteine should be initiated. Furthermore, for patients who present after 10 h, ingested > 105.2 mg/kg or report elevated liver enzyme, supplementary N-acetylcysteine is strongly advised.

## Background

Over the past few decades, the Rumack-Matthew nomogram has been utilized to forecast the danger of hepatotoxicity in patients with acute paracetamol (APAP) poisoning, decide the requirement for N-acetylcysteine (NAC) treatment, and establish the termination of therapy [1, 2]. Presently, the 100-treatment line (100 µg/mL at 4 h and 25 µg/mL at 12 h post ingestion) is accessible in many countries, including the United Kingdom (UK), and the 150-treatment line (150 µg/mL at 4 h and 37.5 µg/mL at 12 h) is used as the baseline for treatment in many countries, including the United States (US) [2–6].

Nonetheless, in some developing countries, emergency medical centers (EMC) lack the appropriate laboratory equipment to promptly report serum APAP concentrations during patient treatment. Consequently, the commencement and cessation of NAC administration are mostly based on a presumption of the APAP poisoning dose obtained from patients and their caregivers and are reliant on blood chemistry tests and the patient’s clinical symptoms [7–9].

The purpose of this study is to investigate clinical risk factors that can predict the detection of APAP concentration after 15 h of ingestion for the safe termination of NAC treatment when treating patients according to the UK guidelines in an environment where serum APAP levels cannot be reported during treatment.

## Methods

### Study design

Data were retrospectively collected over a 132-month period, spanning from 1 January 2010, to 31 December 2020, from two university hospitals in urban area, where over 60,000 patients are seen annually. The database of poisoned patients is digitally recorded according to a predetermined format by the on-duty personnel during a patient’s hospital visit.

### Study population

The study included patients aged at least 14 years who presented with acute poisoning at the hospital within 15 h of ingesting 75 mg/kg/day or more of APAP, which exceeds the maximum therapeutic dose. Patients were excluded if they had chronic liver disease, were taking coumadin, took two or more supratherapeutic doses (≥ 75 mg/kg) over more than 1 h, poisoned with sustained-release APAP, had received activated charcoal treatment at the EMC, or had missing data on ingested dose, weight or APAP concentration.

### Data collection

Data collected for the study included the patient’s age, sex, weight, total dose and time of ingestion as reported by the patients, guardians or paramedics, time elapsed until visiting the EMC, intention of ingestion, components of concomitant poisoning drugs, increased-risk alcohol consumption, eating disorders such as anorexia nervosa, treatment with NAC and time of treatment initiation and termination, blood chemistry test results, serum APAP concentration over time following ingestion, and clinical outcomes.

### Measurements

Among several doses reported by patients, guardians or paramedics, the highest dose was presumed as the ingested dose. Similarly, the time elapsed until hospital visit was inferred based on the longest reported value. Any concomitant poisoning drugs were recorded based on the information provided by the guardians or paramedics, as well as any remaining medications brought to the EMC, prescriptions or by contacting the hospital that issued the prescription. A history of increased-risk alcohol consumption was defined as consuming more than 14 standard alcohol units per week [10].

The detectable concentration after 15 h of ingestion was defined as a level meeting two criteria: it was above the lowest detectable concentration of outsourced laboratory (0.7 µg/mL), and below the corresponding treatment line at that time point. The toxic concentration after 15 h was also defined as concentration above the corresponding treatment line at the time.

Elevated liver enzyme related to APAP overdose was defined as a 50% or greater increase in alanine aminotransferase (ALT) during the treatment period, while acute hepatotoxicity was defined as an increase in ALT greater than 1,000 IU/L [4, 11], the reason for defining with ALT rather than aspartate transaminase (AST) is because the half-life of ALT is longer, nearly 40 h, than AST.

The time of the serum APAP concentration test was recorded in minutes as stated in the test result report. Test results procured prior to 4 h (240 min) after ingestion were considered invalid and were excluded. The results obtained at or after 4-hour (240-minute) mark were recorded as the first test results. The study protocol was evaluated and approved by Institutional Review Board, College of Medicine, and the need for informed consent was waived due to the nature of the retrospective study.

### Treatment protocol

During the study period, the EMC participating in this research did not have institutional resources to obtain APAP concentration results prior to or during NAC treatment. Thus, the initiation and discontinuation of NAC treatment had to be determined on the basis of the reported ingestion dose, blood chemistry test results, and clinical manifestations; the treatment protocol was as follows.

The criteria for administering NAC during the study period were total intake of 10 g within 24 h or ingestion of 200 mg or more per 1 kg of body weight, ingestion of two or more supratherapeutic doses (≥ 75 mg/kg) over more than 1 h, and indefinite ingestion time. NAC treatment followed a 21-hour intravenous (IV) infusion protocol of 150 mg/kg NAC for 15 min, after 45 min, 50 mg/kg for 4 h and 100 mg/kg for 16 h. One hour before the end of NAC treatment, if patient showed impaired consciousness, jaundice, right upper quadrant pain or if blood chemistry results reported ALT, INR or creatinine increased by 50% or more from the pretreatment results, an additional NAC 100 mg/kg infusion treatment was administered for 16 h, and continued until clinical symptoms disappeared, deterioration in blood chemistry test results stopped, and ALT was < 1,000 IU/L [4, 8, 12, 13].

### Key outcome measures

Blood samples to measure APAP levels were taken at least three times, including every 4 h after ingestion and one hour before completing NAC treatment. As the participating EMC lacked clinical laboratory facilities capable of reporting APAP levels in time, the results were later confirmed by sending samples to external specialized clinical laboratories. The lowest detectable concentration of APAP after dilution in the aforementioned institutions was 0.7 µg/mL, and the diagnostic test systems used by the institutions were Cobas^®^ 8000 and Cobas^®^ Integra 400 plus (Roche Diagnostics, Mannheim, Germany), respectively.

### Statistical analysis

For continuous variables, the mean value was calculated if it followed a normal distribution; otherwise, the median value was measured. For categorical variables, the proportion was calculated. The ratios and distributions of variables were compared using the χ^2^ and Kruskal-Wallis test among three groups: patients with undetected serum APAP concentrations after 15 h of ingestion, those with detectable concentrations below the treatment line after 15 h, and those with toxic concentration above the treatment line after 15 h. The statistical significance was reevaluated by post-hoc analysis.

The correlation between risk factors known to predict NAC treatment, such as intentionality of overdose, total ingested dose, ingested dose per unit body weight, acute starvation, malnutrition (low albumin level) and increased-risk alcohol consumption and the detectable and toxic APAP concentration 15 h after ingestion was evaluated using multinomial logistic regression analysis, odds ratios (ORs) and 95% confidence intervals (CIs) were estimated. The area under the receiver operating characteristic curve (AUC) was calculated for risk factors (SPSS version 22.0, IBM Corp., Armonk, NY, USA).

## Results

### Study population

**Fig. 1:**
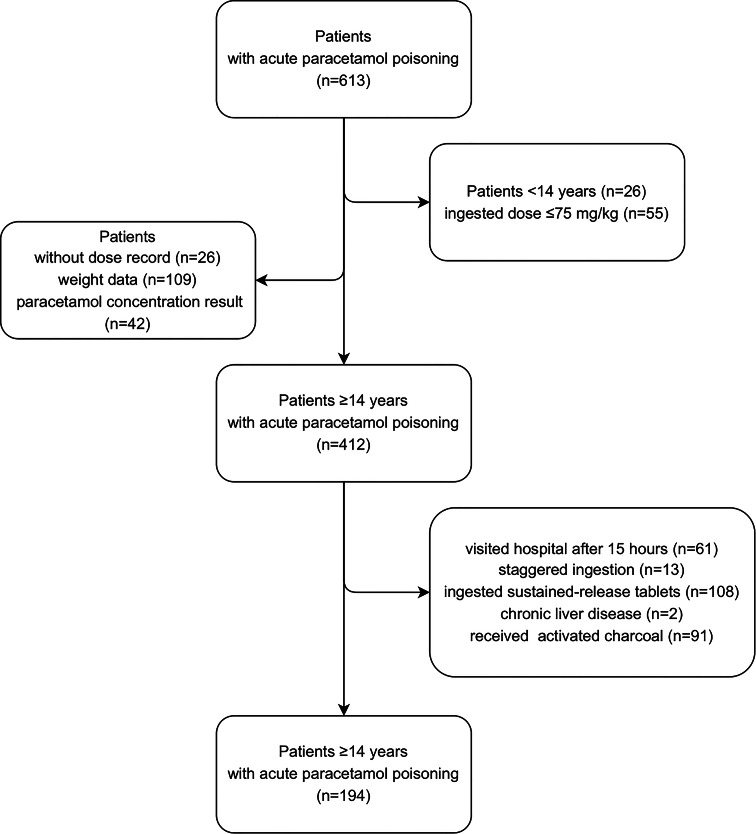
The process of determining the research subjects: patients ≥ 14 years with acute paracetamol poisoning presenting the emergency medical centers

During the study period, a total of 613 patients with acute APAP poisoning visited the hospital. Among them, 26 patients who were under the age of 14 and 55 patients who ingested a dose < 75 mg/kg were excluded. Twenty-six patients who missed the dose in the medical record, 109 patients who did not have weight data available, and 42 patients who did not have APAP concentration results were also precluded. Additionally, 61 patients who visited the hospital more than 15 h after overdose, 13 patients who took two or more doses ≥ 75 mg/kg over more than 1 h, 108 patients who ingested sustained-release tablets, 2 patients with chronic liver disease, 91 patients who received activated charcoal treatment at the EMC were excluded from the study. Therefore, a total of 194 patients were included in the primary analysis (Fig. 1).

Thirty-one patients were male (16.0%), with a median age of 21 years (interquartile range [IQR] 17–32), and 184 patients deliberately ingested APAP (94.8%). The median dose per unit weight of the patients was 152.8 mg/kg (IQR 100.0-234.0). One-hundred and forty-nine patients took other substances with APAP (76.8%), of whom 39 patients took drugs that decreased gastrointestinal motility or induced liver enzymes (20.1%): those included scopolamine, pheniramine, chlorpheniramine, diphenhydramine, methylphenidate, cetirizine, levocetirizine, codeine, dihydrocodeine, dextromethorphan, levodopa, and carbidopa (Table 1).

**Table 1:** Demographic and clinical characteristics of the patients based on serum APAP detection after 15 h from poisoning

	Total	Not detected after 15 h (*n* = 157)	Detectable after 15 h (*n* = 30)	Toxic concentration after 15 h (*n* = 7)	*p*-value
Gender, Male	31 (16.0)	25 (15.9)	5 (16.7)	1 (14.3)	0.591
Age (year)	21 (17–32)	21 (17-32.5)	23 (18–31)	16 (15–31)	0.137
Intentionality	184 (94.8)	149 (94.9)	29 (96.7)	6 (85.7)	0.399
Weight (kg)	56.0 (50.0–65.0)	56.0 (50.0-64.5)	57.0 (48.0–65.0)	54 (43–66)	0.689
Total ingested dose (g)	9.0 (5.6–13.0)	8.8 (5.1–12.7)	10.2 (8.0–22.0)	8.0 (6.5–9.7)	0.074
Ingested dose per kilogram of weight (mg/kg)	152.8 (100.0-234.0)	150.0 (91.6-222.2)	177.7 (132.3–350.0)	166.6 (138.8-190.4)	0.070
Time from ingestion to presentation (minute)	218 (89–431)	200 (82–380)	239 (113–467)	847 (669–871)	< 0.001^c^
Acute starvation	13 (6.7)	12 (7.6)	1 (3.3)	0 (0.0)	0.213
Increased-risk alcohol consumption	12 (6.2)	9 (5.7)	3 (10.0)	0 (0.0)	0.513
Co-ingestion^a^	39 (20.1)	32 (20.4)	7 (23.3)	0 (0.0)	0.327
N-acetylcysteine treatment	158 (81.4)	125 (79.6)	27 (90.0)	6 (85.7)	0.163
Albumin (g/dL)	4.5 (4.2–4.8)	4.6 (4.3–4.8)	4.3(3.8–4.6)	4.1 (3.7–4.5)	0.011^c^
Alanine aminotransferase (IU/L)^b^	14 (10–21)	14 (10–20)	14 (9–28)	17 (10–27)	0.938
Elevated liver enzyme	15 (7.7)	9 (5.7)	6 (20.0)	0 (0.0)	0.132

Variables are expressed as n (%) or median (interquartile range)

^a^ Co-ingestion: overdose with substances that delayed gastric emptying or induced hepatic enzymes

^b^ Alanine aminotransferase: peak level of alanine aminotransferase during treatment

^c^ Post-hoc analysis was performed using Dunn's test with Bonferroni correction for multiple comparisons

During the study period, 87 patients (44.8%) ingested at least 10 g or 200 mg/kg of APAP, and all received NAC treatment. Seventy patients (36.1%) showed concentrations above the 100-treatment line in the first APAP concentration test, with a median time from ingestion to the first test of 449 min (IQR, 271–615).

A total of 7 patients exhibited serum APAP levels under the treatment line on the first test but serum levels above the line on the second test (line-crossers). Three of these patients had no other risk factors for delayed toxicity, apart from the concomitant intake of diphenhydramine, which is known to cause decreased gastrointestinal motility. Among them, a 17-year-old female patient who presented to the hospital 6 h after ingesting 750 mg/kg showed acute hepatotoxicity (peak ALT 6825 IU/L, peak INR 3.87) but was discharged without any sequelae after 4 additional NAC doses of 100 mg/kg of weight (the third bag of NAC).

A total of 37 patients (19.0%) showed detectable and toxic serum APAP concentrations after 15 h of ingestion. Among 30 patients with detectable concentrations, 2 patients exhibited elevated liver enzyme and 4 patients showed acute hepatotoxicity. One of the four patients was the aforementioned patient with delayed toxicity, and the other three were patients who had ingested 333 mg/kg and visited the hospital 13 h later (peak ALT 6819 IU/L, peak INR 1.88); ingested 500 mg/kg and visited the hospital 1 h later (peak ALT 6631 IU/L, peak INR 2.95); and ingested 555 mg/kg and visited the hospital 9 h later (peak ALT 4550 IU/L, peak INR 2.16). All three patients were discharged without any complication after 3, 4, and 2 additional NAC doses of 100 mg/kg. However, none of the seven patients who exhibited toxic concentration after 15 h showed elevated liver enzyme or hepatotoxicity.

### The correlation between risk factors to predict NAC treatment and APAP concentration after 15 h of ingestion

Performing multinomial logistic regression analysis, we investigated whether known risk factors for the need for NAC could predict the detectable and toxic APAP concentration 15 h after ingestion. After post-hoc analysis, serum albumin was excluded from the logistic analysis (adjusted significance between the groups: *p* = 0.100, 0.810, 0.065).

The time elapsed to presentation was measured in minutes, exhibited positive skewness and the patients who presented to the EMC > 15 h after ingestion were previously excluded from the study. The data truncation could have caused a distortion of the distribution of time variable. Since such bias may attenuate the correlation coefficients and effect sizes (odds ratios), the variable of time to presentation was log-transformed and included in the regression analysis.

Initially, no significant predictors were identified between the patients with undetected concentrations after 15 h and those with detectable concentration. In contrast, elapsed time from ingestion to EMC presentation was found to be a significant independent variable in predicting the toxic concentration 15 h after ingestion compared with the undetected serum levels (OR = 1908.536, 95% CI 12.854–283373.365, *p* = 0.003) (Table 2). The AUC for predicting toxic APAP concentration 15 h after ingestion using elapsed time was 0.969 (95% CI 0.934–0.989, *p* < 0.001). The sensitivity and specificity of 612 min cut-off value were 100.0% (95% CI 59.0-100.0) and 92.5% (95% CI 87.8–95.8).

**Table 2:** Multinomial logistic regression analysis for the detection of APAP concentration after 15 h from poisoning

	The patients with detectable concentration (*n* = 30) versus undetected concentration (*n* = 157)		The patients with toxic concentration (*n* = 7) versus undetected concentration (*n* = 157)	
	OR (95% CI)	*p*-value	OR (95% CI)	*p*-value
Time from ingestion to presentation [log(minute)]	1.322 (0.835–2.093)	0.233	1908 (12-283373)	0.003

*OR* Odds ratio, *CI* Confidence interval

## Discussion

A total of 26 and 109 patients did not have information available regarding the ingested dose and weight, respectively, and were excluded from the study. It appeared that these patients had ingested a very low dose, as they were not administered NAC treatment. Based on the median population weight of 56.0 kg, an estimated consumed APAP of 85 patients of them at a dose of < 75 mg/kg. Moreover, among the 42 patients with missing APAP concentration data, 32 had ingested APAP at a dose of < 75 mg/kg. Therefore, it is possible that more patients who had ingested a relatively small dose were excluded from the analysis, leading to an overestimation of the frequency of concentrations above the 100-treatment line in the first APAP concentration test and the detection of APAP concentrations 15 h after ingestion.

**Fig. 2:**
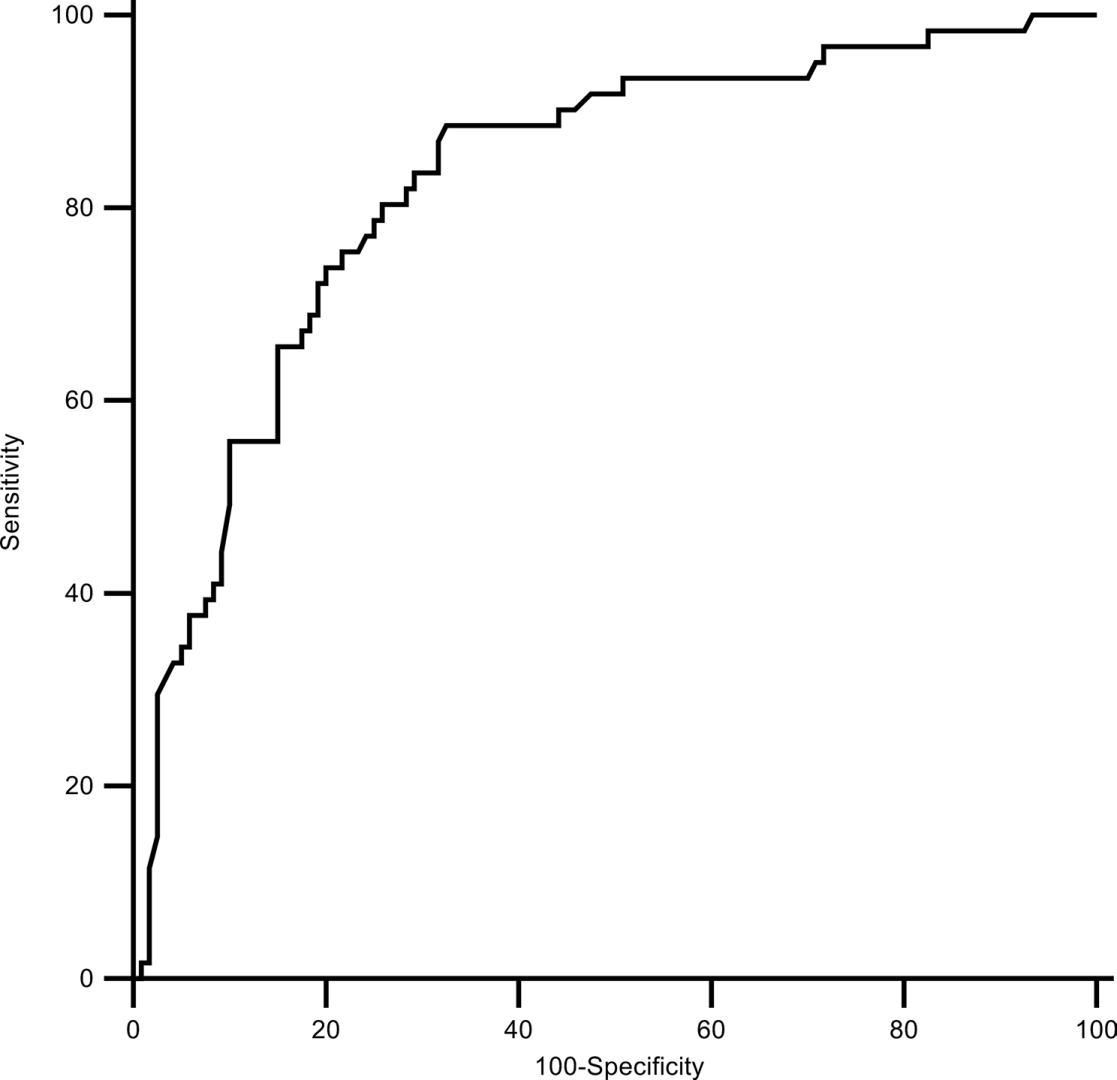
The receiver operating characteristic curve of the ingested dose per unit weight for predicting the concentration above the 100-line in the first test

Three out of 70 patients (4.3%) with concentrations above the 100-treatment line in the first test exhibited hepatotoxicity. The AUC of the ingested dose per unit weight for predicting the concentration above the 100-line was 0.828 (95% CI 0.767–0.878, *p* < 0.001). The sensitivity and specificity of the 150 mg/kg cut-off value were 88.6% (78.7–94.9) and 67.7% (58.8–75.9) (Fig. 2), and the results were consistent with previous studies [2, 4, 6, 9, 14].

The AUC of the time elapsed before visiting the hospital for predicting toxic concentration after 15 h was 0.969 with 612 min cut-off value. In 2004, the Food and Drug Administration (FDA) recommended intravenous NAC treatment within 8 to 10 h after APAP ingestion for acute poisoning patients, and patients who visited the hospital beyond this time were considered “late presenters” with a high risk of the concentration above the treatment line and hepatotoxicity. Therefore, the 10-hour cut-off value revealed in this study is consistent with the recommendations and results of previous studies [2, 4, 6, 15, 16].

The significance of the time variable has long been established. Both the Rumack-Matthew nomogram and the nomogram currently recommended by the UK’s Commission on Human Medicines (CHM) establish treatment criteria based on serum concentrations within 4 to 15-hour. Many researchers, beginning with Prescott et al., have consistently emphasized the importance of assessment and treatment decisions within 15 h [17–20]. Considering these points, our team determined that the UK guideline by which all patients who ingested ≥ 75 mg/kg, maximum therapeutic dose are treated with NAC and use the “100-treatment line” could provide us an effective method for ensuring high sensitivity for the prediction of toxic concentration 15 h after ingestion.

Out of the 30 patients with detectable concentrations below the treatment line after 15 h, 6 patients showed elevated liver enzyme or acute hepatotoxicity, but none of 7 patients who exhibited toxic concentration after 15 h showed elevated liver enzyme or hepatotoxicity. In the subgroup analysis limited to patients with undetected and detectable APAP concentration after 15 h (*n* = 187), ingested dose per weight and elevated liver enzyme were found to be significant predictors (OR = 2.118 and 4.458) (Table 3). The AUC of ingested dose per weight was 0.633, 105.2 mg/kg cut-off value with sensitivity 90.0% and specificity 33.1%. This finding indicates that, even when patients present early to the EMC after ingestion, the ingested dose per weight and elevated ALT should be considered supplementary clinical factors for identifying patients with potential hepatotoxic risk.

**Table 3:** Logistic regression analysis for the detectable APAP concentration after 15 h from poisoning (*n* = 187)

	Univariate (*n* = 187)		Multivariate (*n* = 187)	
	OR (95% CI)	*p*-value	OR (95% CI)	*p*-value
Ingested dose per kilogram of weight [log(mg/kg)]	2.130 (1.191–3.811)	0.011	2.118 (1.192–3.764)	0.011
Albumin (g/dL)	0.531 (0.269–1.049)	0.069	N/A	N/A
Elevated liver enzyme	4.111 (1.342–12.593)	0.013	4.458 (1.364–14.571)	0.013

*OR* Odds ratio, *CI* Confidence interval

This study was an observational retrospective study, which may have led to selection bias and data input errors if patient information was missing or medical records were incomplete. Additionally, the sample size in this study was small (*n* = 194), with a wide range of CIs in the statistical results, and the nonnormal distribution may limit the generalizability of the study findings. Given that the median ingested dose per unit weight of the study subjects was comparatively low (152.8 mg/kg), it is possible that the frequency of detecting APAP concentrations above the 100-treatment line and the frequency of detecting APAP blood concentrations after 15 h were relatively low.

## Conclusion

In an environment where serum APAP concentrations cannot be measured during treatment following the UK guidelines, if patients are aged 14 years or older and present to the EMC within 15 h of an acute APAP poisoning, in all patients who have ingested more than 75 mg/kg, the maximum therapeutic dose, NAC treatment should be initiated without delay. Furthermore, for patients who present to the EMC after 10 h from ingestion, ingest more than 105.2 mg/kg or report elevated liver enzyme, supplementary NAC treatment is strongly advised.

## Data Availability

The datasets used and/or analyzed during the study are available from the corresponding author upon reasonable request.

## Abbreviations

APAP: Paracetamol
AUC: Area Under the Receiver Operating Characteristic Curve
CI: Confidence Interval
EMC: Emergency Medical Centers
FDA: The Food and Drug Administration
IQR: Interquartile Range
IV: Intravenous
NAC: N-Acetylcysteine
OR: Odds Ratio
UK: United Kingdoms
US: United States
